# Perception of Older Adults Toward Smartwatch Technology for Assessing Pain and Related Patient-Reported Outcomes: Pilot Study

**DOI:** 10.2196/10044

**Published:** 2019-03-26

**Authors:** Todd Matthew Manini, Tonatiuh Mendoza, Manoj Battula, Anis Davoudi, Matin Kheirkhahan, Mary Ellen Young, Eric Weber, Roger Benton Fillingim, Parisa Rashidi

**Affiliations:** 1 Department of Aging and Geriatric Research University of Florida Gainesville, FL United States; 2 Department of Health Outcomes and Biomedical Informatics University of Florida Gainesville, FL United States; 3 Department of Computer and Information Science and Engineering University of Florida Gainesville, FL United States; 4 Department of Biomedical Engineering University of Florida Gainesville, FL United States; 5 Department of Occupational Therapy University of Florida Gainesville, FL United States; 6 Department of Community Dentistry and Behavioral Science University of Florida Gainesville, FL United States

**Keywords:** smartwatch, focus group, ecological momentary assessment (EMA), patient-reported outcomes (PRO)

## Abstract

**Background:**

Chronic pain, including arthritis, affects about 100 million adults in the United States. Complexity and diversity of the pain experience across time and people and its fluctuations across and within days show the need for valid pain reports that do not rely on patient’s long-term recall capability. Smartwatches can be used as digital ecological momentary assessment (EMA) tools for real-time collection of pain scores. Smartwatches are generally less expensive than smartphones, are highly portable, and have a simpler user interface, providing an excellent medium for continuous data collection and enabling a higher compliance rate.

**Objective:**

The aim of this study was to explore the attitudes and perceptions of older adults towards design and technological aspects of a smartwatch framework for measuring patient report outcomes (PRO) as an EMA tool.

**Methods:**

A focus group session was conducted to explore the perception of participants towards smartwatch technology and its utility for PRO assessment. Participants included older adults (age 65+), with unilateral or bilateral symptomatic knee osteoarthritis. A preliminary user interface with server communication capability was developed and deployed on 10 Samsung Gear S3 smartwatches and provided to the users during the focus group. Pain was designated as the main PRO, while fatigue, mood, and sleep quality were included as auxiliary PROs. Pre-planned topics included participants’ attitude towards the smartwatch technology, usability of the custom-designed app interface, and suitability of the smartwatch technology for PRO assessment. Discussions were transcribed, and content analysis with theme characterization was performed to identify and code the major themes.

**Results:**

We recruited 19 participants (age 65+) who consented to take part in the focus group study. The overall attitude of the participants toward the smartwatch technology was positive. They showed interest in the direct phone-call capability, availability of extra apps such as the weather apps and sensors for tracking health and wellness such as accelerometer and heart rate sensor. Nearly three-quarters of participants showed willingness to participate in a one-year study to wear the watch daily. Concerns were raised regarding usability, including accessibility (larger icons), notification customization, and intuitive interface design (unambiguous icons and assessment scales). Participants expressed interest in using smartwatch technology for PRO assessment and the availability of methods for sharing data with health care providers.

**Conclusions:**

All participants had overall positive views of the smartwatch technology for measuring PROs to facilitate patient-provider communications and to provide more targeted treatments and interventions in the future. Usability concerns were the major issues that will require special consideration in future smartwatch PRO user interface designs, especially accessibility issues, notification design, and use of intuitive assessment scales.

## Introduction

About 100 million adults in the United States are affected by chronic pain, including pain caused by arthritis, costing US $560-$635 billion annually [1]. Pain is a complex experience [2] that varies across time and people [3,4]. Recent research on pain in arthritis patients has shown that pain fluctuates significantly both across and within days [3]. Traditionally, researchers and practitioners have relied on patients’ recall to assess pain, as well as to track and evaluate pain management routines [5]. While still a convenient method, many recent studies point to memory errors and distortions that influence pain recall [6-9]. For example, the “peak-end effect” causes the more recent experiences to have an especially strong influence on recall [10], and the “duration neglect” results in a tendency to ignore periods without pain [11]. To provide valid patient-reported outcomes (PROs) on pain that do not rely on patients’ long-term recall capability, researchers have used various ecological momentary assessment (EMA) approaches such as paper-and-pencil and electronic diaries [6,12], Twitter feeds [13], and smartphone apps [14,15]. EMA methods ask individuals to provide systematic daily diaries of their experiences at random occasions. These approaches can provide finer resolution and possibly more valid assessments, while also providing the ability to examine the fluctuations and variation of pain over time. The use of digital EMA tools can be especially important for enhancing the accuracy of assessments in older adults, who are more likely than younger adults to experience memory lapses [16].

Smartphones have increased in popularity as convenient digital EMA tools for real-time assessments [14,15]. This trend even expands in older adults, with 70% of the population currently owning a smartphone. While this is encouraging for the feasibility of using smartphone research–related apps [17], it has not carried forward into smartwatches [18]. Older adults may lack the requisite knowledge and skills for effectively using a smartwatch for EMA and for monitoring other health-related characteristics. In this study, we examined the perception and attitude of older adults towards smartwatch technology for capturing pain PROs. We specifically used the Samsung Gear S3 smartwatch. It is less expensive than a smartphone, highly portable, and discrete due to its sleek design resembling a regular watch. These factors promote higher compliance. A smartwatch also has a much simpler user interface than a smartphone, and due to its enhanced portability, a smartwatch provides an excellent medium for continuous data collection.

We hypothesized that since a smartwatch can be worn all day, this will potentially result in a higher compliance rate compared to a smartphone. A smartwatch, coupled with the embedded sensors including accelerometer, global positioning system (GPS), ultraviolet (UV), and heart rate sensor can provide additional information such as physical activity intensity and duration, location, UV exposure, and heart rate. Previous EMA interventions based on basic watch-type EMA tools for assessing fatigue have been reported to be successful at characterizing the temporal changes of fatigue [19], demonstrating the potential for momentary assessments. We assessed the attitudes of older adults towards smartwatch technology for capturing pain PRO measures in a focus group to guide hardware and software development and our long-term studies. A preliminary version of the PROMPT (Patient Reported Outcome of Mood, Pain, and faTigue) app was developed, along with the server infrastructure, which were provided to the participants during a demo session. The focus group discussions and suggestions were summarized and analyzed to assess the potential of smartwatch technology for PRO assessment and to guide future developments for use in older adults.

## Methods

### Study Population

We recruited 20 older adults aged 65-89 years, and 19 of them participated in the focus group. The inclusion criteria included age ≥65 years and diagnosis of unilateral or bilateral symptomatic knee osteoarthritis. Some of the exclusion criteria included failure or inability to provide informed consent; significant cognitive impairment, defined as a known diagnosis of dementia; and being unable to communicate because of severe hearing loss or speech disorder (see Multimedia Appendix 1 for eligibility criteria). A convenient sample of older adults was identified through posting flyers at University of Florida’s Institute on Aging research and patient clinics and direct mailings to age-eligible participants from approved registries. Each participant received compensation of a US $50 gift card. The focus group protocol was approved by the University of Florida Institutional Review Board.

### Smartwatch App and Server Framework

**Figure 1:**
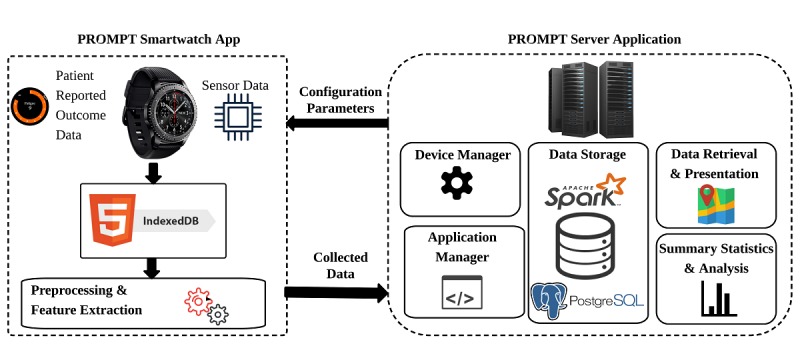
The PROMPT (Patient Reported Outcome of Mood, Pain, and faTigue) framework: the smartwatch app and the server application.

The PROMPT framework is made up of two components: (1) the server software and (2) the smartwatch app. This integrated framework is designed and developed to perform several tasks including remote data collection, storage, retrieval, and analysis. Figure 1 depicts the main component of the system. The PROMPT framework was developed at the University of Florida to enable real-time capturing of patient-generated information, including wearable sensor data, along with self-report PRO assessments as described previously [20].

**Figure 2:**
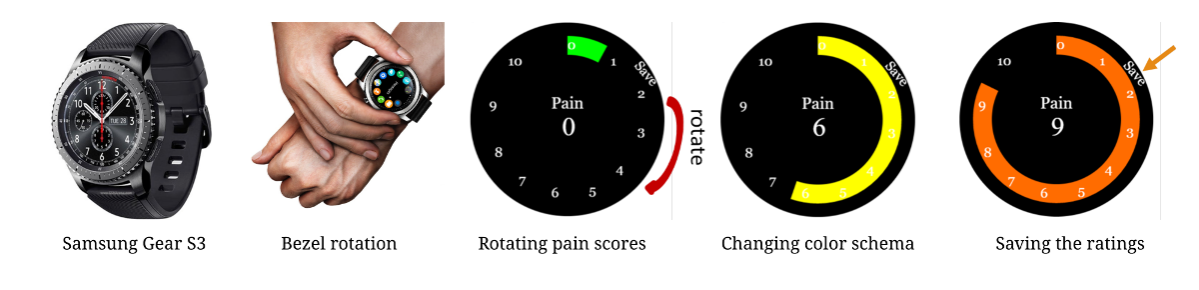
The Samsung Gear S smartwatch used in the PROMPT (Patient Reported Outcome of Mood, Pain, and faTigue) study. Ratings are entered by rotating the bezel to select pain ratings. The color schema also changes as the ratings are increased or decreased. Ratings are saved by pressing the top button (physical button), located on top of the bezel.

The PROMPT app was developed to show assessment notification every 4 hours by asking users to enter their current pain, fatigue, and mood assessments. No messages were shown during the nighttime to avoid any sleep disruptions. Messages were provided only from 8 a.m.-8 p.m. Sleep quality was programmed to be assessed every morning with a message randomly displayed between 8 a.m.-12 p.m. Using the PROMPT interface, the assessment ratings could be easily entered by rotating a bezel and could be saved by pressing a button located on top of the bezel (Figure 2). While we have presented only the pain assessment screen (Figure 2), similar screens have been developed for assessing fatigue, mood, and sleep quality. We used the Numerical Pain Rating Scale (NRS) [21] for pain assessment by showing pain intensity on a scale of 0-10. Other auxiliary PROs including mood, fatigue, and sleep were shown similarly using a numerical scale of 0-10 [22,23]. All these scales except for the sleep quality designated 10 as the worst possible outcome (ie, highest pain level, highest fatigue level, or the most negative mood).

**Figure 3:**
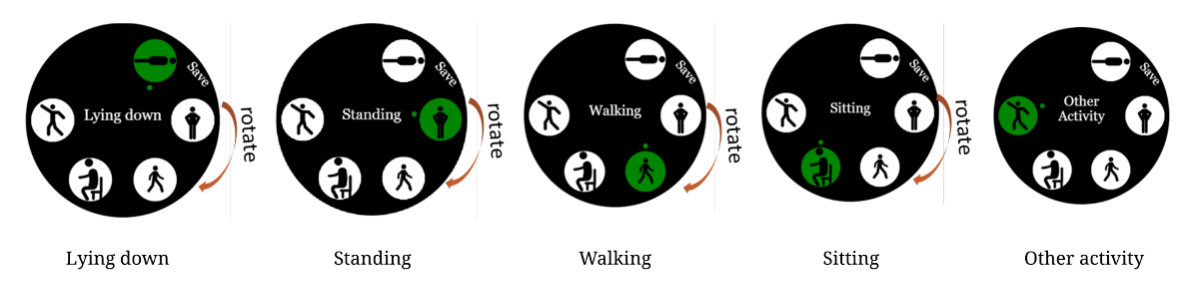
Users can choose activities by rotating the bezel.

The same bezel rotation and saving mechanism was also used to capture current user activities (Figure 3). Our current list of activities included lying down, standing, walking, sitting, and “other activities” representing other possible activities such as gardening and exercise.

### Focus Group Set-Up

The focus group was conducted by a team consisting of a moderator and 2 assistant moderators. The focus group formation and content analysis were guided by memo writing, qualitative sampling, and metacoding [24-26]. The moderator used a semistructured interview to present information with a goal of promoting uninhibited dialogue and nonjudgmental feedback. Research assistants took notes of verbatim quotes. The assistant moderators also observed and documented participants’ expressions and reactions. No audio recording was performed for privacy reasons and to provide a more inviting discussion atmosphere. Both assistant moderators helped facilitate the discussions. One of the assistants took notes on a large easel pad, clearly visible to all participants, while also posting participants’ notes on the easel using Post-it notes provided to the participants at the beginning of the session. The other assistant moderator took notes on a laptop computer and tallied the number of participants discussing each topic.

The first 30 minutes of the focus group was dedicated to introducing the smartwatch technology, explaining the rationale of the study, and showing screenshots of the interface. Then the participants were provided with 10 Gear S3 smartwatches preloaded with the PROMPT app. They were assisted in using the PROMPT app, as necessary. The watch configuration was changed to show notifications every 5 minutes to better allow for exploration of the app in a timely manner. Last, to better capture design preferences, the participants were asked to sketch their own smartwatch face design.

### Focus Group Orientation and Questions

**Figure 4:**
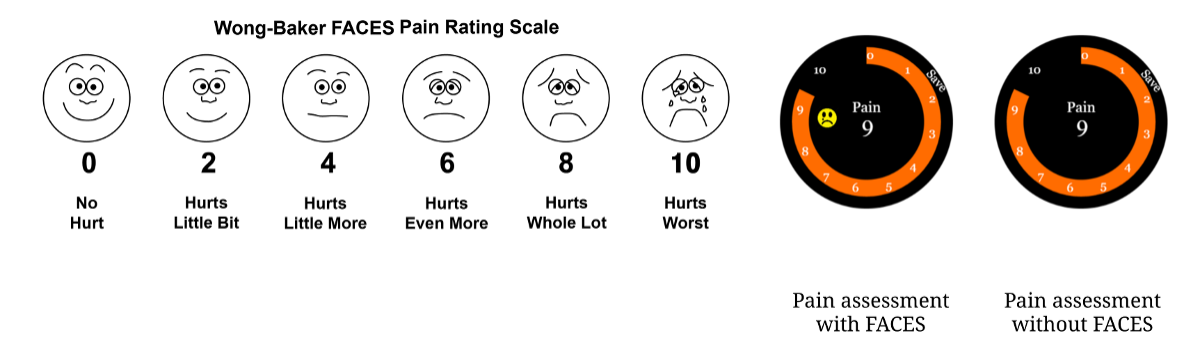
Wong-Baker FACES Pain Rating Scale (left). PRO assessment with and without emoticons. Source: Wong-Baker FACES Foundation.

The focus group was designed to be an open-ended forum, starting with several directed questions. We asked 12 questions that related to the impression of the smartwatch technology and mimicked questions that are traditionally used to evaluate computer app and mobile app interfaces, including the System Usability Scale [27] and Mobile App Rating Scale [28]. These questions were designed to provide feedback on the PROMPT user interface, using a smartwatch for PRO assessment, long-term study logistics, and potential future improvements. While the questions had direct responses, all question included time and discussion for open-ended feedback (Table 1). Most of the questions related to the user interface were based on current PROMPT interface implementation to identify necessary improvements. Alternative scenarios, such as using emoticons on the assessment screens using the Wong-Baker FACES Pain Rating Scale [29], were shown during the presentation (Figure 4).

The rationale for including questions a.1 (watch size) and a.2 (first impression) was to identify the general acceptability of a smartwatch in daily settings, or in a one-year study (questions d.1 and d.2). The rationale for including questions b.1-6 was to assess the existing user interface and identify possible issues and to outline smartwatch interface guidelines for older adults’ population. Finally, questions c.1 and c.2 were included to specifically solicit information on assessing PROs through a smartwatch interface.

### Analysis

Following the focus group, the notes were compiled and summarized by the assistant moderators. Major topics were identified across the discussions by the assistant moderators and were grouped based on the underlying themes. The theme codes were developed based on note data to categorize data into overarching interpretive themes. The codes were then refined to fit data through an iterative summative process [30]. This process continued until themes and properties were easily distinguishable and succinct [30]. Chi-square tests were used to test for differences in proportions in dichotomous variables.

**Table 1:** Focus group questions summarized according to their topic.

Topic	Questions
a. Smartwatch impression	a.1 What is your opinion about watch size and its accessory bands?
	a.2 What is your first impression of the watch itself?
b. PROMPT interface	b.1 Do you like the PROMPT color schema for PRO assessment?
	b.2 Do you like the app flow? Any need for a back button?
	b.3 Would you like to add emoticons to the assessment screen?
	b.4 Do you like the activity icons? Would you prefer icons or text?
	b.5 What type of notification do you prefer to receive, and why?
	b.6 Is the text large enough to read?
c. PRO assessment	c.1 How many times per day would be too burdensome to ask you?
	c.2 Other issues you would like the researchers and doctors to know?
d. Study logistics	d.1 How likely are you to participate in a one-year research study asking you to wear the smartwatch daily for up to a year?
	d.2 What other options would help you to participate?

## Results

### User Statistics

Of the 20 participants who consented to the study, 19 participated in the focus group study. The session lasted about 90 minutes. Table 2 depicts the demographics information of participants. Test of proportion was performed on characteristics among male and female participants for applicable responses.

### Content Analysis

The content analysis revealed several major subtopics and themes under each major topic (Table 1), as shown in Tables 3 and 4. A total of 109 verbatim quotes from participants were coded, and nine of the quotes were considered to be irrelevant. The themes emerged under the four groups of questions (ie, smartwatch impression, PROMPT user interface, PRO assessment, and study logistics). We identified 13 major themes and 48 detailed subthemes.

Theme percentages do not include the tally questions. Some discussion items were included under multiple themes. The discussion on user interface options was the most comprehensive (just over half of all the topics discussed), spanning issues from accessibility for visually impaired users to specific details of design. The participants expressed a desire for customization, for example, to choose how to be notified when it is time to enter the PRO assessments (eg, sound, vibration, and music) or to customize the list of activities or medications. Initially, most participants showed interest in using emoticons like the Wong-Baker FACES Pain Rating Scale [29] to guide them during assessment, but after working with the app on the watch, they felt there was no need for emoticons, given the color change during rating. Participants were also asked about issues and possible improvements in the PRO assessment process. Answers included the ability to provide more detailed information, such as indicating fluctuations, activity dependent measures, pain location, and the ability to provide medication usage. Besides existing PROs, participants showed interest in tracking joint stiffness and sleep.

**Figure 5:**
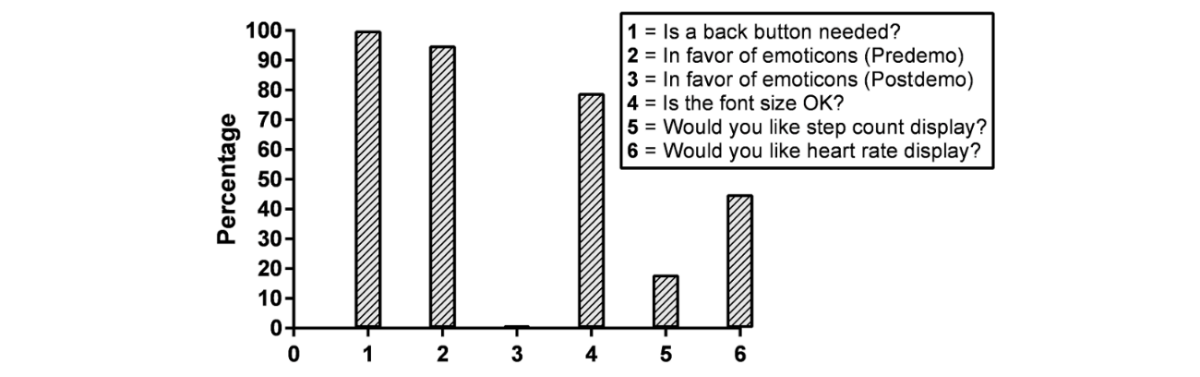
Participant preferences on various user interface issues related to PROMPT (Patient Reported Outcome of Mood, Pain, and faTigue). Bars indicate the percentage of users who responded "Yes".

The participants were asked about several issues regarding the PROMPT app user interface, including the need for emoticons on PRO scales, the use of back button, font size, and displaying additional information such as heart rate or step count (Figure 5). The participants were asked to indicate their response by raising their hand for an affirmative response. The assistant moderators documented the counts.

**Figure 6:**
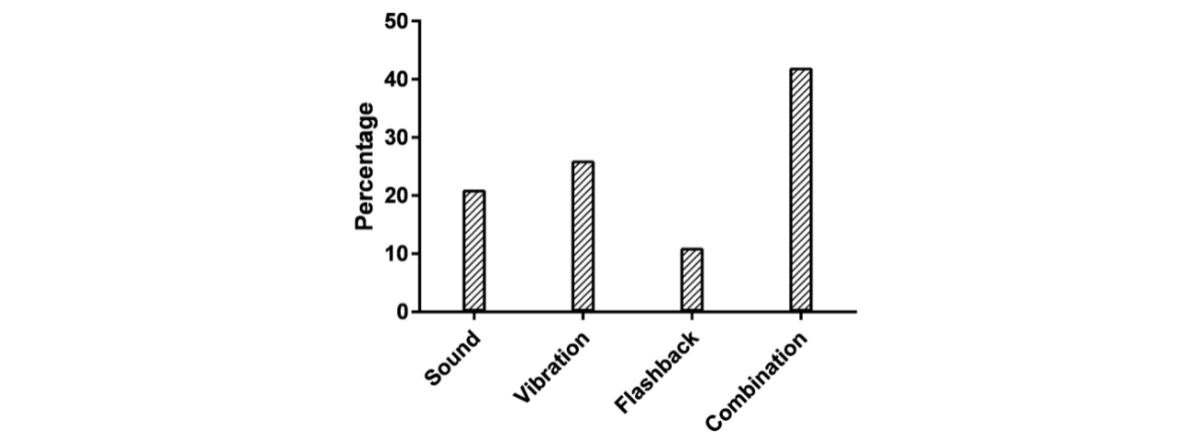
Participant preferences on notifications type.

**Figure 7:**
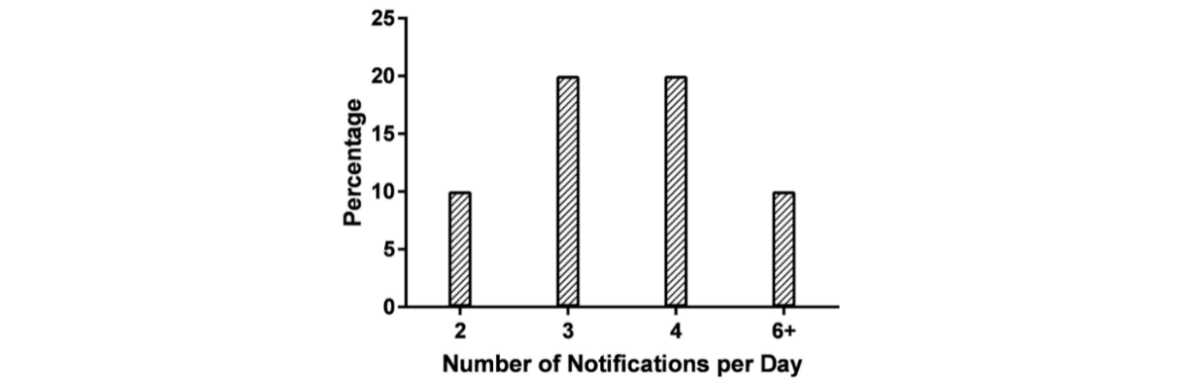
Participant preferences on notifications frequency.

The participants were asked about their notification method of choice (Figure 6) and whether they would prefer sound, vibration, flashing light, or a combination of all. The participants were also asked about preferred number of notifications per day (Figure 7). The PRO assessment discussions led to the comment that EMA might not be able to capture the maximum pain experience during the day, if sampled at certain times. It was suggested that instead of displaying messages for PRO assessments four times a day, it might be better to display the messages three times, while asking for a summary assessment at the end of the day to better capture daily fluctuations. Additionally, 74% (14/19) of the participants mentioned that they would be willing to participate in a one-year study in which they would wear the watch every day. This increased to 89% (17/19) when we clarified that the watch can be worn during domestic and international travel.

**Table 2:** Characteristics of the focus group participants (N=19).

Characteristics	Total	Female	Male	*P* value
Participants, n (%)	19	14 (74)	5 (26)	.01
Age (years), mean (SD)	72.7 (6.1)	72.0 (6.7)	75.5 (5.8)	.22
Access to Wi-Fi, n (%)	17 (89)	—^a^	—	—
Own a smartphone, n (%)	14 (74)	—	—	—
Own a smartwatch, n (%)	1 (5)	1 (7)	0 (0)	1
Active in water, n (%)	4 (21)	—	—	—

^a^Data were not collected per female/male, only collectively.

**Table 3:** Themes and subthemes reported by the focus group participants (percentages are the percent reported with respect to all the other themes).

Topic, themes, and subthemes			n (%)	
**Smartwatch impression (25%)**				
	**Desired functions (32%)**			
		Time display^a^		1 (5)
		Apps^a^		3 (16)
		Water resistance^a^		1 (5)
		Backlight^a^		1 (5)
		Security^d^		1 (5)
	**Desired apps (27%)**			
		Weather^a^		3 (16)
		Email^a^		1 (5)
		Phone^a^		2 (11)
	**Appearance concerns (32%)**			
		Heavy body^b^		2 (11)
		Accessory bands^a^		4 (21)
		Band durability^c^		1 (5)
	**Desired sensors (9%)**			
		Step count^a^, heart rate^a^, GPS^a^		2 (11)
**PROMPT user interface (54%)**				
	**Color schema (12.5%)**			
		Accessibility for color-blind individuals^c^		2 (11)
		Customized color schema^c^		3 (16)
		Mapping colors to mental states^c^		3 (16)
	**Icons (18.7%)**			
		Icon ambiguity^b^		2 (11)
		Expanded list of activities^c^		1 (5)
		Customized list of activities^c^		1 (5)
		Activity intensity^c^		1 (5)
		Emoticons^c^		4 (21)
	**Notifications (33.3%)**			
		Notification preferences^c^		3 (16)
		Disruptive notifications^d^		2 (11)
		Notification type customization^c^		1 (5)
		Context-dependent notifications^c^		1 (5)
		Silent mode^a^		1 (5)
		Number of notifications^d^		7 (37)
		Start time customization^c^		1 (5)
	**Usability and accessibility (27.0%)**			
		Easy setup^a^		4 (21)
		Automatic messages^a^		1 (5)
		Speech input^c^		1 (5)
		Larger font size^c^		3 (16)
		Large icons^c^		3 (16)
		Notification customization for visually or hearing impaired^c^		1 (5)
	**Assessment scales (6.25%)**			
		Scale visual aid^c^		2 (11)
		Neutral value visual aid^c^		1 (5)
	**Flow (2.0%)**			
		Back navigation button^c^		1 (5)
**PRO assessment (18%)**				
	**Capturing pain (50%)**			
		Ability to indicate fluctuation and intermittent pain^c^		2 (11)
		Ability to indicate activity dependent measures^c^		1 (5)
		Ability to indicate pain location^c^		1 (5)
		Weekly or daily summary^c^		1 (5)
		Ability to indicate medication use^c^		3 (16)
	**Other PROs (50%)**			
		Ability to indicate stiffness^c^		1 (5)
		Receiving more positive feedback instead of negative^c^		4 (21)
		Ability to track sleep^c^		3 (16)
**Study logistics (2%)**				
	**Study participation (100%)**			
		Use during travel^c^		1 (5)
		Frequent clinic visit, Impact on personal data plan^d^		1 (5)

^a^Positive existing feature (I liked it).

^b^Undesirable existing feature (I did not like it).

^c^Desired future feature (I would like to see that).

^d^Undesirable/concerning future feature (I would be concerned about that).

**Table 4:** Selected participants’ quotes on discussed themes grouped according to topic.

Topic and subtopic		Example quotes
**Smartwatch impression**		
	Function	“Can you download its apps like on a smartphone?”
	Apps	“I would wear it as it is; it is excellent, but the more apps, the better.”
	Appearance	“I like the extra band, lighter.”
	Sensors	“Can its GPS be used to track if I am at the gym?”
**PROMPT user interface**		
	Color schema	“When it shows my good mood as green, I don’t like it, not my mental model of happiness.”
	Icons	“Standing can represent both washing dishes or cooking.”
	Notifications	“My hearing is bad, and I might be active and might not see it.”
	Usability & accessibility	“Voice-activated recording might be helpful to record details of activities.”
	Assessment scales	“For feeling down, is the scale going up or down?”
	Flow	“I would like an erase or back button when I make a mistake.”
**PRO assessment**		
	Capturing pain	“I have intermittent pain walking for five minutes, then no pain, coming and going.”
	Other patient-reported outcomes	“It is important to emphasize when you are feeling good, feeling up. To emphasize fatigue, it is negative, and it is going to be measured in a negative way.”
**Study logistics**		
	Study participation	“How would the watch affect my data plan usage?”

## Discussion

### Principal Findings

A review of the literature shows the lack of systematic evaluation of smartwatch technology among older adults. While several recent studies have developed smartwatch apps for fall detection [31], mood assessment [32], or gait estimation [33], there has been limited research [32] on using smartwatch technology for PRO assessment in the general population and more specifically among older adults.

This study allowed us to explore the attitudes and perceptions of older adults towards smartwatch technology, specifically for PRO assessment. Most participants in our study expressed enthusiasm for wearing the smartwatch, despite its weight and lack of several desired features, which points to the potential feasibility of using such a device in long-term studies or daily settings. In general, while it has been shown that older adults are less likely to use new technology compared with younger adults [34], there is ample evidence that they also desire interaction with new technologies to remain active and engaged with society [35]. In a recent framework, Lee and Coughlin identified 10 factors that affect how technology is adopted by older adults, including perceived value, usability, affordability, accessibility, technical support, social support, emotion, independence, experience, and confidence [36]. Our results are consistent with these factors and with previous studies on the use of technology among older adults [37-39], indicating an interest in adopting new technology given perceived usefulness and potential benefits.

Several previous studies also have found that anxiety is positively correlated with age while self-efficacy is negatively correlated, resulting in lower self-confidence and higher anxiety in older adults when facing new technology [34,40]. As Lee and Coughlin point out [41], it is important to build an intuitive design to enhance self-confidence among older adults. Our focus group results demonstrate that a smartwatch provides a significant degree of familiarity by resembling a regular watch, thus facilitating knowledge transfer and overcoming the learning barriers, possibly building confidence in older adults’ ability to use this new technology [41].

In general, the participants perceived the smartwatch technology and its use for PRO assessment as an empowering tool as it allows them to provide real-world symptomology to caregivers. This is particularly true for chronic pain, which is often highly variable [42]. They also indicated that a simple interface, technical support, and clear instructions are needed to tackle the technological barriers, which is consistent with other studies [36,43]. App interface customization also was a recurring theme throughout the focus group discussions, pointing to the need to tailor the app to users’ individual needs and preferences and to accommodate hearing and visual impairment, further underlining the need for usability and accessibility.

**Figure 8:**
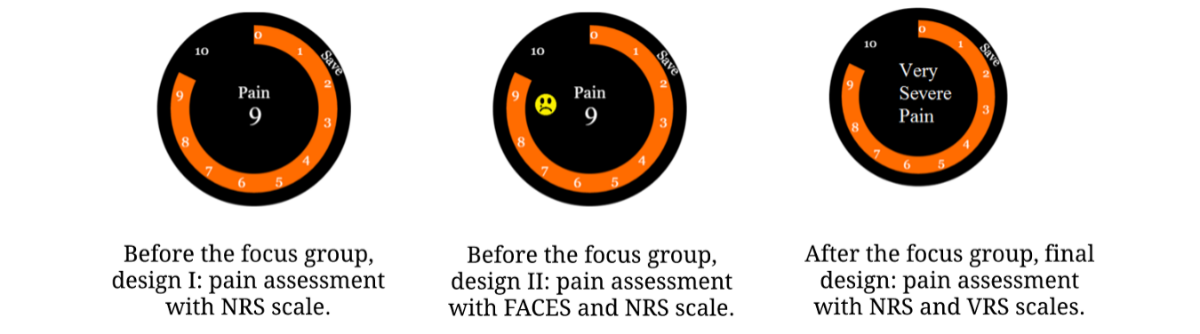
Different pain assessment scales used before and after the focus group. NRS: Numerical Pain Rating Scale; VRS: Verbal Pain Rating Scale.

We found that participants’ mental models of assessment scales can greatly impact how they assess their outcomes (“For feeling down, is the scale going up or down?”). For example, initially we used NRS [44] for pain assessment by representing pain intensity on a scale 0-10 (Figure 8). Based on our focus group discussion, we changed our design to reflect a combination of NRS and the Verbal Pain Rating Scale [45] (Figure 8) to avoid confusion and to better allow the participants to map the smartwatch scale to their mental scale. As discussed before, interestingly, the participants did not think it was necessary to use the Wong-Baker FACES Pain Rating Scale [29] to guide them during rating (Figure 8). Similar verbal scales are used in our refined design for mood, fatigue, and sleep assessment. We adopted existing verbal scales such as a modified version of the Visual Analogue Mood Scale [46] for mood assessment. We also changed some of the wording such as “feeling down” to “mood” to reflect a more neutral sentiment and to avoid negative thought reinforcement.

We also found that, in general, the touchscreen interface on the smartwatch was difficult to operate by some older adults due to the small size of icons, as well as their decreased motor resolution and coordination, as observed in previous studies on older adults with smartphones [47]. Most participants preferred using the bezel rotation and the physical button pressing. Based on this feedback, our redesigned app uses only these mechanisms for interacting with the app.

The participants also expressed interest in several future features, most notably the capability to keep their health care provider in the loop through a health care provider portal or through Electronic Health Records integration. They also showed interest in a patient Web portal for viewing their collected data in more detail on a larger screen device. Connectivity to other smart devices such as smart scales was also discussed by participants. 

Finally, an emergency option, the ability to call 911 or relatives in case of emergency, was on top of their future desired features.

### Limitations

Though our results point to interesting insights, our study had several limitations. Our focus group participants were recruited locally and might not represent the broader population of older adults. This is reflected in a higher rate of smartphone ownership among our participants compared to the national smartphone ownership in the older adult population. The results also are based on a single focus group session following limited interaction with the technology, and different results could emerge if feedback was obtained after wearing and using the device for an extended period. Finally, we studied the smartwatch technology primarily in the context of pain assessment and participants reporting knee pain. These results might differ if the focus group was conducted on the use of smartwatch for different applications or when targeting populations with different medical histories. Nonetheless, our results point to the feasibility of using smartwatches for PRO assessment in older adults, and they offer invaluable insights for improving the current interface and technology.

### Future Research

Future studies are needed to explore the perceptions of older adults toward such PRO assessment interfaces and how their perceptions change after wearing the smartwatch for a given period. We plan to use our PRO assessment app for quantifying and comparing PROs such as pain among different populations of older adults in real-life settings. Future work will also compare the use of PRO assessment tools on different devices, including smartphones, tablets, and smartwatches, to better identify the differences among such mediums. Finally, there is a need to integrate patient-generated information with routine care data in a format that is useful to care providers.

### Conclusions

Our study examined the acceptability for using smartwatch technology as a PRO assessment in older adults in a focus group setting. Our questions on participants’ willingness to take part in a one-year study, as well as questions on the appeal of smartwatch size and interface design, reflect the potential feasibility of using a smartwatch in long-term studies or daily settings. Usability and intuitive design, personalization, and accessibility were found to be important for adopting and using PROMPT smartwatch technology. The choice of different PRO assessment methods (eg, visual vs verbal scales) was also found to impact how older adults use smartwatch technology for reporting their pain, mood, fatigue, and sleep quality. Finally, the participants expressed interest in the ability to observe these assessments in more detail on a Web portal and to be able to share them with their health care providers. These findings can be used to guide the future smartwatch software design, as well as to guide developing new EMA methods for PRO assessment.

## Appendix

Multimedia Appendix 1 Inclusion and exclusion criteria.

## Abbreviations

EMA: ecological momentary assessment
GPS: global positioning system
NRS: Numerical Pain Rating Scale
PRO: patient-reported outcomes
PROMPT: Patient Reported Outcome of Mood, Pain, and faTigue
UV: ultraviolet

## References

[1] Gaskin DJ, Richard P (2012). The economic costs of pain in the United States. J Pain.

[2] Craig KD (2015). Social communication model of pain. Pain.

[3] Ho A, Ashe MC, DeLongis A, Graf P, Khan KM, Hoppmann CA (2016). Gender Differences in Pain-Physical Activity Linkages among Older Adults: Lessons Learned from Daily Life Approaches. Pain Res Manag.

[4] Fillingim RB (2017). Individual differences in pain: understanding the mosaic that makes pain personal. Pain.

[5] Rothman M, Burke L, Erickson P, Leidy NK, Patrick DL, Petrie CD (2009). Use of existing patient-reported outcome (PRO) instruments and their modification: the ISPOR Good Research Practices for Evaluating and Documenting Content Validity for the Use of Existing Instruments and Their Modification PRO Task Force Report. Value Health.

[6] Stone AA, Broderick JE, Schneider S, Schwartz JE (2012). Expanding options for developing outcome measures from momentary assessment data. Psychosom Med.

[7] Daoust R, Sirois M-J, Lee JS, Perry JJ, Griffith LE, Worster A, Lang E, Paquet J, Chauny J-M, Émond M (2017). Painful Memories: Reliability of Pain Intensity Recall at 3 Months in Senior Patients. Pain Res Manag.

[8] Bąbel P (2014). The effect of affect on memory of pain induced by tooth restoration. Int Dent J.

[9] Bąbel P, Bajcar E A, Śmieja M, Adamczyk W, Świder K, Kicman P, Lisińska N (2018). Pain begets pain. When marathon runners are not in pain anymore, they underestimate their memory of marathon pain--A mediation analysis. Eur J Pain.

[10] Chajut E, Caspi A, Chen R, Hod M, Ariely D (2014). In pain thou shalt bring forth children: the peak-and-end rule in recall of labor pain. Psychol Sci.

[11] Guse A, Wunderlich A, Weiss B, Möller S (2016). Duration neglect in multi-episodic perceived quality. 8th International Conference on Quality of Multimedia Experience (QoMEX 2016).

[12] Stone AA, Broderick JE (2007). Real-time data collection for pain: appraisal and current status. Pain Med.

[13] Nascimento TD, DosSantos MF, Danciu T, DeBoer M, van Holsbeeck H, Lucas SR, Aiello C, Khatib L, Bender MC A, Zubieta J-K, DaSilva AF, UMSoD (Under)Graduate Class Of 2014 (2014). Real-time sharing and expression of migraine headache suffering on Twitter: a cross-sectional infodemiology study. J Med Internet Res.

[14] Stinson JN, Jibb LA, Nguyen C, Nathan PC, Maloney AM, Dupuis LL, Gerstle JT, Alman B, Hopyan S, Strahlendorf C, Portwine C, Johnston DL, Orr M (2013). Development and testing of a multidimensional iPhone pain assessment application for adolescents with cancer. J Med Internet Res.

[15] Thomas JG, Pavlovic J, Lipton RB, Roth J, Rathier L, O'Leary KC, Buse DC, Evans EW, Bond DS (2016). Ecological momentary assessment of the relationship between headache pain intensity and pain interference in women with migraine and obesity. Cephalalgia.

[16] Rullier L, Atzeni T, Husky M, Bouisson J, Dartigues JF, Swendsen J, Bergua V (2014). Daily life functioning of community-dwelling elderly couples: an investigation of the feasibility and validity of Ecological Momentary Assessment. Int J Methods Psychiatr Res.

[17] (2018). Pew Research Center.

[18] Seifert A, Schlomann A, Rietz C, Schelling HR (2017). The use of mobile devices for physical activity tracking in older adults' everyday life. Digit Health.

[19] Yoshiuchi K, Cook DB, Ohashi K, Kumano H, Kuboki T, Yamamoto Y, Natelson BH (2007). A real-time assessment of the effect of exercise in chronic fatigue syndrome. Physiol Behav.

[20] Nair S, Kheirkhahan M, Davoudi A, Rashidi P, Wanigatunga AA, Corbett DB, Manini TM, Ranka S (2016). ROAMM: A software infrastructure for real-time monitoring of personal health.

[21] Ferreira-Valente MA, Pais-Ribeiro JL, Jensen MP (2011). Validity of four pain intensity rating scales. Pain.

[22] Alghadir AH, Anwer S, Iqbal A, Iqbal ZA (2018). Test-retest reliability, validity, and minimum detectable change of visual analog, numerical rating, and verbal rating scales for measurement of osteoarthritic knee pain. J Pain Res.

[23] Nadarajah M, Mazlan M, Abdul-Latif L, Goh HT (2017). Test-retest reliability, internal consistency and concurrent validity of Fatigue Severity Scale in measuring post-stroke fatigue. Eur J Phys Rehabil Med.

[24] Liamputtong P (2011). Focus group methodology: Principle and practice.

[25] Glaser BG, Strauss AL (1999). The Discovery Of Grounded Theory: Strategies For Qualitative Research.

[26] Krueger RA, Casey MA (2014). Focus groups: A practical guide for applied research.

[27] Brooke J, Jordan PW, Thomas B, Weerdmeester BA, McClelland AL (1996). SUS-A quick and dirty usability scale. Usability evaluation in industry.

[28] Stoyanov SR, Hides L, Kavanagh DJ, Zelenko O, Tjondronegoro D, Mani M (2015). Mobile app rating scale: a new tool for assessing the quality of health mobile apps. JMIR Mhealth Uhealth.

[29] Wong DL, Baker CM (2001). Smiling faces as anchor for pain intensity scales. Pain.

[30] Creswell JW (2006). Qualitative Inquiry And Research Design: Choosing Among Five Approaches.

[31] Deutsch M, Burgsteiner H (2016). A Smartwatch-Based Assistance System for the Elderly Performing Fall Detection, Unusual Inactivity Recognition and Medication Reminding. Stud Health Technol Inform.

[32] Bachmann A, Klebsattel C, Schankin A, Riedel T, Beigl M, Reichert M, Santangelo P, Ebner-Priemer U (2015). Leveraging smartwatches for unobtrusive mobile ambulatory mood assessment. Adjunct Proceedings of the 2015 ACM International Joint Conference on Pervasive and Ubiquitous Computing and Proceedings of the 2015 ACM International Symposium on Wearable Computers.

[33] Nemati E, Suh YS, Motamed B, Sarrafzadeh M (2016). Gait velocity estimation for a smartwatch platform using kalman filter peak recovery.

[34] Czaja SJ, Charness N, Fisk AD, Hertzog C, Nair SN, Rogers WA, Sharit J (2006). Factors predicting the use of technology: findings from the Center for Research and Education on Aging and Technology Enhancement (CREATE). Psychol Aging.

[35] Kurniawan S (2008). Older people and mobile phones: A multi-method investigation. International Journal of Human-Computer Studies.

[36] Lee C, Coughlin Jf (2014). PERSPECTIVE: Older Adults' Adoption of Technology: An Integrated Approach to Identifying Determinants and Barriers. J Prod Innov Manag.

[37] Moons KGM, Altman DG, Reitsma JB, Ioannidis JPA, Macaskill P, Steyerberg EW, Vickers AJ, Ransohoff DF, Collins GS (2015). Transparent Reporting of a multivariable prediction model for Individual Prognosis or Diagnosis (TRIPOD): explanation and elaboration. Ann Intern Med.

[38] Peek STM, Aarts S, Wouters E, van Hoof J, Demiris G, Wouters E (2017). Can smart home technology deliver on the promise of independent living? A critical reflection based on the perspectives of older adults. Handbook of Smart Homes, Health Care and Well-Being.

[39] Liu L, Stroulia E, Nikolaidis I, Miguel-Cruz A, Rios Rincon A (2016). Smart homes and home health monitoring technologies for older adults: A systematic review. Int J Med Inform.

[40] Chung JE, Park N, Wang H, Fulk J, McLaughlin M (2010). Age differences in perceptions of online community participation among non-users: An extension of the Technology Acceptance Model. Computers in Human Behavior.

[41] Lee C, Coughlin JF (2014). PERSPECTIVE: Older Adults' Adoption of Technology: An Integrated Approach to Identifying Determinants and Barriers. J Prod Innov Manag.

[42] Zakoscielna KM, Parmelee PA (2013). Pain variability and its predictors in older adults: depression, cognition, functional status, health, and pain. J Aging Health.

[43] Hill R, Betts LR, Gardner SE (2015). Older adults’ experiences and perceptions of digital technology: (Dis)empowerment, wellbeing, and inclusion. Computers in Human Behavior.

[44] McCaffery M, Pasero C (1999). Pain: Clinical Manual.

[45] Kliger M, Stahl S, Haddad M, Suzan E, Adler R, Eisenberg E (2015). Measuring the Intensity of Chronic Pain: Are the Visual Analogue Scale and the Verbal Rating Scale Interchangeable?. Pain Pract.

[46] van Rijsbergen GD, Bockting CLH, Berking M, Koeter MWJ, Schene AH (2012). Can a one-item mood scale do the trick? Predicting relapse over 5.5-years in recurrent depression. PLoS One.

[47] Page T (2014). Touchscreen mobile devices and older adults: a usability study. IJHFE.

