# Supplementary material for: Perception of Older Adults Toward Smartwatch Technology for Assessing Pain and Related Patient-Reported Outcomes: Pilot Study
Source: JMIR Mhealth Uhealth. 2019 Mar 26;7(3):e10044. doi: 10.2196/10044 (PMC6454335; doi:10.2196/10044)
Supplement: Multimedia Appendix 1 [file mhealth_v7i3e10044_app1.pdf]

Table Inclusion and exclusion criteria
**Inclusion Criteria**<br>• Men and women age ≥65 years<br>• unilateral or bilateral symptomatic knee OA
**Exclusion criteria**<br>• Failure or inability to provide informed consent<br>• Lives in a nursing home; persons living in assisted or independent housing are not excluded<br>• Significant cognitive impairment, defined as a known diagnosis of dementia, or a Mini-Mental State Exam (MMSE) score <24<br>• Unable to communicate because of severe hearing loss or speech disorder<br>• Neurological conditions that are causing impaired muscle function or mobility (may include stroke with residual paresis, paralysis, neuropathy, Parkinson disease, or multiple sclerosis)<br>• Severe rheumatologic or orthopedic diseases, e.g., awaiting joint replacement, known active inflammatory or autoimmune disease (e.g., rheumatoid arthritis, lupus, Crohn's disease, HIV)<br>• Terminal illness with life expectancy less than 12 months<br>• Severe pulmonary disease, requiring either steroid pills or injections or the use of supplemental oxygen<br>• Other significant co-morbid disease that in the opinion of the field center PI would impair ability to participate in the trial, e.g., renal failure on hemodialysis, severe psychiatric disorder (e.g., bipolar, schizophrenia), excessive alcohol use (>14 drinks per week); drug addiction; treatment for cancer (radiation or chemotherapy) within the past 1 year; or other conditions<br>• Lives outside of the study site
**Temporary exclusion criteria**<br>• Stroke, hip fracture, hip or knee replacement, or spinal surgery within past 4 months<br>• Participation in another intervention trial within 3 months; participation in an observational study may be permitted
